# Lifestyle Habits and Risk of Cardiovascular Mortality in Menopausal Women with Cardiovascular Risk Factors: A Retrospective Cohort Study

**DOI:** 10.3390/jcdd11090287

**Published:** 2024-09-16

**Authors:** Adriana Lopez-Pineda, Cristina Soriano-Maldonado, Vicente Arrarte, Francisco Sanchez-Ferrer, Vicente Bertomeu-Gonzalez, Juan Miguel Ruiz-Nodar, Jose A. Quesada, Alberto Cordero

**Affiliations:** 1 GRINCAVA Research Group, Clinical Medicine Department, Miguel Hernandez de Elche University, 03550 Alicante, Spain; adriana.lopezp@umh.es (A.L.-P.); c.soriano@umh.es (C.S.-M.); f.sanchez@umh.es (F.S.-F.); vbertog@gmail.com (V.B.-G.); ruiz_jmi@gva.es (J.M.R.-N.); jquesada@umh.es (J.A.Q.); acorderofort@gmail.com (A.C.); 2 Network for Research on Chronicity, Primary Care and Health Promotion (RICAPPS), 03550 Alicante, Spain; 3 Primary Care Research Center, Miguel Hernández University, 03550 San Juan de Alicante, Spain; 4 Primary Care Department of Muchamiel, 03110 Alicante, Spain; 5 Cardiology Department, Dr. Balmis de Alicante University Hospital, 03010 Alicante, Spain; 6 Pediatrics Department, San Juan de Alicante University Hospital, 03550 San Juan de Alicante, Spain; 7 Cardiology Department, Benidorm Clinical Hospital, 03501 Benidorm, Spain; 8 Spanish Cardiovascular Research Network (CIBERCV), 28029 Madrid, Spain; 9 Cardiology Department, IMED Hospital, 03203 Alicante, Spain

**Keywords:** heart disease risk factors, postmenopause, women, life style, mortality, diabetes, dyslipidaemia, hypertension

## Abstract

Current cardiovascular prevention guidelines emphasise considering sex, gender, and gender identity in risk assessment. This study evaluated the impact of lifestyle habits and chronic diseases on cardiovascular mortality risk in women over 50 with high vascular risk and developed a predictive model for menopausal women with cardiovascular risk factors. A retrospective cohort study used data from the 2011 Spanish National Health Survey and the national death register, focusing on menopausal and postmenopausal women without prior cardiovascular events but with at least one major risk factor. Participants were followed for up to 10 years, assessing mortality from circulatory system diseases and other causes. Exposure variables included socio-demographics, lifestyle habits, health status, self-perceived health, health service use, and pharmacological treatments. Of the 21,007 respondents, 3057 women met the inclusion criteria. The 10-year cumulative incidence of mortality from circulatory causes was 5.9%, and from other causes, 12.7%. Independent predictors of cardiovascular mortality were never consuming legumes, poor self-perceived health, diabetes treatment, lack of physical activity, and older age. Lipid-lowering treatment was protective. The model demonstrated good fit and predictive capacity (C-index = 0.773). This study highlights the significant influence of physical activity, legume consumption, self-perceived health, and specific treatments on cardiovascular mortality risk in menopausal women.

## 1. Introduction

Current cardiovascular prevention guidelines recognise the importance of integrating sex, gender, and gender identity considerations into risk assessment and clinical management of individuals and populations [1]. Perimenopausal women with risk factors are more likely to experience cardiovascular events than men of the same age. In fact, cardiovascular disease is the leading cause of mortality in women, causing up to four times more deaths in women compared to breast cancer [2].

Cardiovascular risk management in women is suboptimal, especially during the menopausal transition, when there is a general decline in sex hormones and thus increased susceptibility to cardiovascular events [3]. There are clear differences in the epidemiology, symptoms, diagnosis, progression, prognosis, and management of cardiovascular risk between men and women. The main risk factors to be controlled in perimenopausal women are hypertension, dyslipidaemia, obesity, and other components of the metabolic syndrome, while avoiding and adequately controlling diabetes [4]. In addition, while traditional cardiovascular risk factors dominate in older age, there are several female-specific risk factors and inflammatory risk variables that influence a woman’s risk at a younger age [5]. Therefore, the menopausal transition has an additional adverse effect on ageing that may require specific attention to ensure an optimal cardiovascular risk profile and quality of life [6]. Our research group has already shown that risk in this population is significantly influenced by lifestyle habits, with factors such as vegetable consumption and physical activity being particularly relevant in this population [7]. Other factors, however, such as dyslipidaemia and obesity, which are strong predictors in men, do not seem to have such a high predictive value in postmenopausal women.

Addressing gender differences in healthcare is very important to prevent cardiovascular events; compared to men, women with ischaemic heart disease are less likely to receive evidence-based treatment and, when they suffer an acute myocardial infarction, are less likely to receive reperfusion [8,9,10,11,12]. In addition, the more male-centred evidence on drug development, due to the under-representation of women in clinical trials for cardiovascular disease, does not help to address these differences [13]. Many cardiovascular risk prediction models exist for the general population [14]; while most do not take into account the differential characteristics of women, several models in the USA have included female-specific risk factors [15,16]. The aim of this study was to determine the influence of lifestyle habits and chronic diseases on the increased risk of cardiovascular mortality in a sample of women aged 50 years or older at high or very high vascular risk, and to construct a specific predictive model for menopausal women with cardiovascular risk factors.

## 2. Materials and Methods

A population-based retrospective observational cohort study was conducted using the 2011 Spanish National Health Survey (ENSE11) and the national database of deaths by cause of death, both provided by the National Institute of Statistics [17]. This study complies with the recommendations of the Declaration of Helsinki and was approved by the Office of Responsible Research of the University (Ref. AUT.DMC.ALP.240323).

The study population was women residing in Spain, of menopausal and postmenopausal age without a previous cardiovascular event and with at least one major cardiovascular risk factor who responded to the ENSE11. Inclusion criteria were: (i) being a woman; (ii) being aged ≥ 50 years; (iii) having answered yes to question G21c of the survey (Has a doctor told you that you have suffered from any of these health problems?) regarding diabetes, high cholesterol and/or high blood pressure; (iv) and not having answered yes to the same question G21c that a doctor has told you that you have suffered from acute myocardial infarction, other heart disease or embolism, cerebral infarction or cerebral haemorrhage.

The ENSE11 was conducted in the one-year period between July 2011 and June 2012, on 21,007 adult (15 years and older) residents in Spain using stratified tri-stage sampling.

Participants were followed up by probabilistic cross-checking of the ENSE11 with the national death register from 2011 to 2021 inclusive, a maximum of 10 years.

The response variables were mortality from circulatory system disease (ICD10: I00-I99) and death from other causes, recorded during follow-up. The exposure variables were:

- socio-demographics: age, region of residence, social class based on the reference person’s occupation [18], country of birth, marital status, level of education, and net monthly household income;
- lifestyle habits: tobacco consumption, frequency of exposure to smoky environments, alcohol consumption, hours of sleep per day, type of physical activity performed in the main daily activity, frequency of physical activity in leisure time, fruit consumption, vegetable consumption, legume consumption, dairy consumption, sweets consumption, fast food consumption, and dental hygiene;
- health status: body mass index (BMI), use of hearing aid, use of glasses or contact lenses, any chronic or long-term illness, course or history of high blood pressure, varicose veins, osteoarthritis, chronic neck pain, chronic low back pain, allergy, asthma, chronic obstructive pulmonary disease, diabetes mellitus, stomach ulcer, urinary incontinence, high cholesterol, cataracts, chronic skin problems, constipation, cirrhosis, migraine, haemorrhoids, osteoporosis, thyroid problems, malignant tumours, chronic depression, chronic anxiety or permanent injuries caused by an accident, and mental health;
- self-perceived health and health-related quality of life: perceived health status in the last twelve months, health status using the visual analogue scale (VAS) of the EQ-5D-5L quality of life questionnaire, limitation in activities of daily living due to health problem for at least the last six months, and bed rest more than half a day for health reasons in the last two weeks;
- use of health services: hospital admission in the last 12 months, day hospital admission in the last 12 months, emergency care in the last 12 months, primary care consultation care in the last month, specialist consultation care in the last month, physiotherapy consultation care in the last year, psychology consultation care in the last year, X-rays in the last year, CT scans or scans in the last year, ultrasound scans in the last year, MRI scans in the last year, and flu vaccination in the last campaign;
- pharmacological treatments: cholesterol-lowering drugs, diabetes drugs, and blood pressure drugs.

The response options for each variable are shown in Table S1 of the supplementary material.

### Statistical Analysis

The treatment of missing values was performed as follows: For some qualitative variables, a maximum of 0.5% missing values were observed (16 cases), and a simple imputation process was performed by the highest frequency of the variable, according to the mortality response variable (cardiovascular, other causes or alive) of each person. For the quantitative variable VAS of the EQ5D questionnaire, 61 missing values (1.9%) were detected and imputed by the mean value according to the mean value of each category of the mortality response variable. Descriptive analysis was performed for all qualitative variables by calculating frequencies, and mean values and standard deviation were calculated for quantitative variables. To estimate the magnitudes of the 10-year cardiovascular mortality risks, multivariate Cox models of competing risks between cardiovascular mortality and mortality from other causes were fitted using the approach of Putter et al. [19] applied by Moore [20] and by a previous study by this same research team [7]. This approach allows the incorporation of time-dependent variables with competing risks and takes into account the complex sample design of the survey with a weighted analysis. Hazard Ratio (HR) and their 95% confidence intervals (95%CI) were estimated, crude age-adjusted models were constructed for each explanatory variable separately, and an optimal multivariate model was constructed. To arrive at an optimal model based on the principle of parsimony, a forward stepwise variable selection based on the Akaike information criterion was performed. The proportional hazards hypothesis of the model was tested. If any variable violated the proportional hazards assumption, a time-dependent term was introduced by adding an interaction term between the variable and follow-up time (variable x T), where T represents the follow-up time and the variable is coded in dummy form. Goodness-of-fit was tested using the likelihood ratio test (LRT). As an indicator of the predictive ability of the model, the C-index and its 95% CI were calculated. The model was built on a random test sample composed of 70% of the original sample, and validation was performed on the random test sample with 30% of the original size. From the optimal model, a 10-year cardiovascular mortality risk score scale was constructed following the approach described by Sullivan et al. [21]. We estimated mean survival times from the Cox model, applying the approach of Bender et al. [22] through the Gompertz distribution with simulations of the uniform distribution between zero and one. To obtain greater stability in the estimates, we replicated the process 10,000 times. In this way, we estimated a mean survival time for each person in the sample. Finally, we obtained the mean survival time for each risk profile (sum of points on the risk scale) with its 95% CI. To obtain estimates representative of the Spanish population, complex sampling was taken into account by using as a weighting factor the survey elevation factor divided by its mean, thus obtaining weights centred on its mean [23]. The analyses were carried out using R v.4.3.1.

## 3. Results

**Figure 1:**
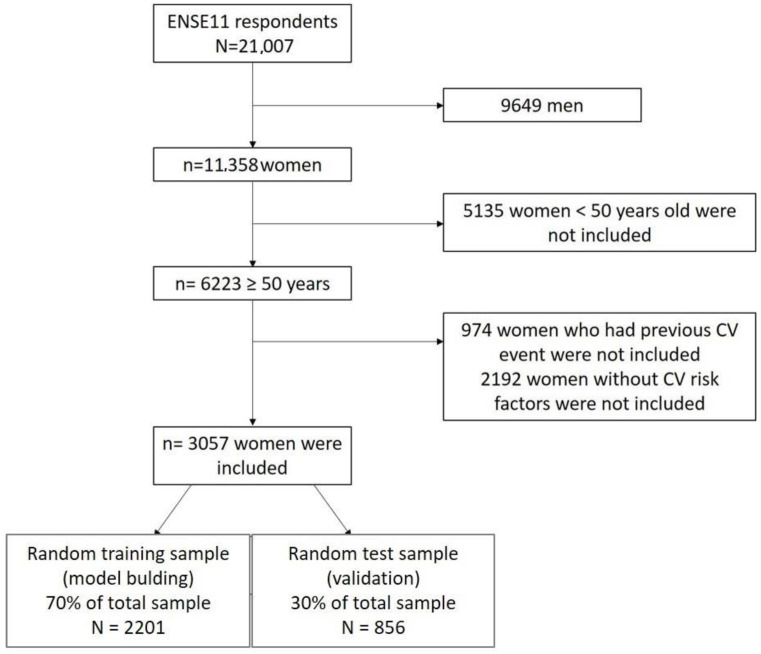
Flow chart of the study.

Of the total 21,007 respondents, 3057 women meeting the inclusion criteria were included (Figure 1) with a mean age of 67.9 years (SD 10.6 and range 50–102 years), 28.5% being older than 75 years. The cumulative incidence of 10-year mortality from circulatory system causes was 5.9% (180 cases), and from other causes 12.7% (389 cases). Table 1 shows the characteristics of the sample: 56.6% were overweight or obese, 80% had never smoked, 39.0% reported sitting most of the time in their daily life, 51.5% reported no physical activity in leisure time, 58.9% consumed vegetables daily, 25.8% consumed legumes more than 3 times a week, 17.7% said their general self-perceived health was poor or very poor, 7.3% said they had COPD, 19.6% said they had depression, and 16.9% said they had anxiety. Nine per cent had been admitted to hospital in the last year, and 57.9 per cent were being treated for hypertension, 37.4 per cent for dyslipidaemia, and 16.3 per cent for diabetes. The visual analogue scale on general health had a mean score of 66.0 (SD 19.5) where 100 points is the best possible health. Table S1 presents the full description of the sample using population proportion estimates.

Table S2 shows the adjusted risks of cardiovascular death estimated by competing risk models for each explanatory variable with a crude adjustment for age. Table 2 shows the optimal multivariate model fit and shows that the independent predictors of cardiovascular mortality were never consuming legumes (HR 6.814) or consuming them less than two times a week (HR 3.768); having fair (HR 2.29), poor (HR 2.90), or very poor (HR 3.20) self-perceived health; being treated for diabetes (HR 2.01); not doing any leisure-time physical activity (HR 1.48); and older age (HR 1.156 for each year of increase). Receiving lipid-lowering treatment (HR 0.63) was a protective factor for cardiovascular mortality. The main daily activity of sitting most of the day has a time- dependent risk of cardiovascular death, so that at the beginning of follow-up it is high (HR 3.27) and decreases during follow-up according to EXP function (1.185–0.0098 × T), with T being the months of follow-up between 1 and 120 months. The model fits the data well (LRT Chi2 821.8 *p* < 0.001), presenting an acceptable predictive capacity in the testing sample (n = 856) with C-index 0.773 (95%CI 0.740–0.805).

Table 3 shows the risk scale constructed from the multivariate model, according to which, for example, a 66-year-old woman who spends most of her time sitting during her main daily activity does some leisure-time physical activity, consumes legumes daily, has a self-perceived fair health, and is not on lipid-lowering treatment but is treated for diabetes has a score of five, giving a cardiovascular mortality risk of 0.4% and a mean survival time of 5.5 years.

## 4. Discussion

The present study shows that the cardiovascular mortality risk of a woman of menopausal age (≥50 years) with at least one cardiovascular risk factor (diabetes, dyslipidaemia, or hypertension) and no previous cardiovascular disease is mainly determined by her physical activity, legume consumption, self-perceived health, and treatment for diabetes and dyslipidaemia.

Lifestyle and diet are at the core of cardiovascular prevention [24], and their implementation is included in all clinical practice guidelines for the treatment of cardiovascular risk factors and secondary prevention [1]. Adherence to a balanced diet and physical activity have been directly linked to the development of atherosclerosis [25]. However, in recent decades a worsening in all elements of lifestyle and diet has been observed in developed countries, reflected in increased obesity and sedentary lifestyles [26]; moreover, some of these changes have been more marked among women, especially smoking [27]. The results of our study emphasise the key role of sedentary lifestyles and vegetable consumption in the debut of cardiovascular disease in the group of women with the highest incidence and mortality from this cause.

Regarding the consumption of legumes, a recent meta-analysis on the general population found no significant association between legume consumption and cardiovascular disease mortality (HR: 1.01, 95% CI: 0.94, 1.08) [28]. This meta-analysis, which included data from over 500,000 participants, suggests that while legumes may contribute to reduced all-cause mortality and stroke risk, their effect on CVD mortality in the general population remains uncertain. It is important to note that the population in this meta-analysis was general, whereas our study focuses specifically on postmenopausal women with cardiovascular risk factors. This population may respond differently due to hormonal changes that occur during menopause, which could amplify the protective effects of legume consumption. Therefore, our study adds to the growing body of evidence, highlighting the importance of considering specific populations when assessing dietary interventions for cardiovascular prevention.

Moreover, this analysis of the ENSE11 reflects the protective role of lipid-lowering treatment; indeed, it is the variable conferring the greatest protection against the development of ischaemic heart disease. Although this health survey does not specifically record the active ingredients that each woman was receiving, multiple national registries have shown that statins constitute the total practice of lipid-lowering treatments; this is consistent with other international studies [29,30]. Control of dyslipidaemia, specifically reduction in low-density lipoprotein cholesterol (LDL-C) values, correlates directly and linearly with reduction in the incidence of ischaemic heart disease; furthermore, this has been observed in observational, Mendelian randomisation or multidrug studies [31]. In contrast, being treated for diabetes was associated with an increased risk, and this could be related to poor metabolic control of diabetes, but especially to the use of drugs that do not reduce the incidence of cardiovascular complications. Since the publication of the EMPAREG Outcomes study in 2015 [32] and the LEADERS study [33], SGLT2 inhibitors and GLP1 analogues have been positioned as priority drugs for improving cardiovascular prognosis in patients with diabetes. Since the survey data used for our study predate the publication of these studies and the commercialisation of the drugs, we believe that our results would reflect the prognosis of women with diabetes treated with the drugs available at that time.

Although cardiovascular risk factors such as smoking and alcohol consumption, as well as protective factors like fruit and vegetable intake, are well-established in the general population, they did not emerge as significant predictors in this cohort of postmenopausal women. This may be due to the specific characteristics of the sample, where other variables like physical inactivity, diabetes, and dyslipidaemia carry more weight in this high-risk population. These findings highlight the need for further studies exploring these factors in populations with different risk profiles.

Limitations of this study that could affect the interpretation and generalisability of the results are as follows: the use of retrospective data from the ENSE11 may introduce biases inherent to the retrospective nature of the information collected; the use of self-reported data on lifestyle habits and health conditions raises the possibility of recall bias and subjectivity, which could affect the accuracy of the associations identified; the absence of specific data on salt/sodium consumption, which is known to be an important dietary factor influencing cardiovascular risk, limits our ability to evaluate its potential confounding role in relation to other dietary components; and finally, the imputation of missing data and the construction of a specific predictive model for menopausal women with cardiovascular risk factors are aspects that, although approached with methodological rigour, could limit the applicability and generalisability of the findings to other populations. It is important, therefore, to interpret the results with caution and to conduct future research to address these limitations in order to strengthen the scientific evidence in this field.

The results of this study are only applicable to women aged 50 years or older, without prior cardiovascular events, and with at least one major cardiovascular risk factor. The postmenopausal transition amplifies certain risks in women, reinforcing the need for sex-specific prevention strategies. While these factors are well-established in the general population, further research is needed to understand their unique impacts on women during the menopausal transition and to refine prevention strategies accordingly.

In conclusion, this study underlines the importance of healthy lifestyle habits and proper management of chronic diseases to reduce the risk of cardiovascular mortality in menopausal women with risk factors. It highlights the need for sex-sensitive approaches to cardiovascular prevention, promoting active lifestyles and balanced diets, along with effective treatments for chronic conditions.

## Figures and Tables

**Table 1:** General characteristics of the sample (n = 3057).

Variables		n	%
Group age	50–64	1272	41.6
	65–74	913	29.9
	75–84	664	21.7
	>84	208	6.8
Body Mass Index	Normal	788	25.8
	Overweight	1021	33.4
	Obesity	709	23.2
	NS/NC	539	17.6
Country of birth	Spain	2853	93.3
	Foreign	204	6.7
Level of education	University students	195	6.4
	FP	182	6.0
	Bachelor	204	6.7
	Secondary	928	30.3
	Primary or less	1548	50.6
Tobacco use	Never	2456	80.4
	Ex-smoker	294	9.6
	Smoker	306	10.0
Sleeping hours per day	>9h/day	214	7.0
	7–9 h/day	1835	60.0
	<7 h/day	1008	33.0
Main daily activity	Sitting most of the time	1191	39.0
	Standing for most of the time	1628	53.3
	Walking with some weight	209	6.8
	Tasks with effort	29	0.9
Leisure physical activity	Sedentary	1574	51.5
	Occasional physical activity	1227	40.1
	Frequent physical activity	140	4.6
	Sports training	116	3.8
Vegetable consumption	On a daily basis	1800	58.9
	>3 times/week	943	30.9
	1–2 times/week	244	8.0
	<1 time/week	52	1.7
	Never or almost never	18	0.6
Consumption of legumes	On a daily basis	57	1.9
	>3 times/week	731	23.9
	1–2 times/week	1847	60.4
	<1 time/week	339	11.1
	Never or almost never	83	2.7
Self-perceived health	Very good	190	6.2
	Good	1151	37.7
	Fair	1173	38.4
	Bad	432	14.1
	Very bad	111	3.6
COPD	Yes	222	7.3
Depression	Yes	599	19.6
Anxiety	Yes	518	16.9
Hospital admission last year	Yes	276	9.0
Treatment of hypertension	Yes	1771	57.9
Dyslipidaemia treatment	Yes	1145	37.4
Diabetes treatment	Yes	500	16.3

**Table 2:** Cox multivariate model for cardiovascular mortality with competing risks of death from other causes.

		Betas	Error	HR	95%CI	*p*-Value
Main daily activity	Some activity			1		
	Sitting most of the time	1.1852	0.322	3.272	(1.740–6.150)	<0.001
Physical activity leisure	Some activity			1		
	Sedentary	0.3939	0.145	1.483	(1.116–1.970)	0.006
Consumption of legumes	On a daily basis			1		
	≥3 times/week	1.2328	0.615	3.431	(1.028–11.453)	0.045
	≤2 times/week	1.3266	0.606	3.768	(1.149–12.359)	0.029
	Never or almost never	1.9190	0.664	6.814	(1.854–25.039)	0.004
Self-perceived health	Very good			1		
	Good	0.5850	0.401	1.795	(0.818–3.939)	0.145
	Fair	0.8290	0.394	2.291	(1.058–4.960)	0.035
	Bad	1.0637	0.409	2.897	(1.300–6.459)	0.009
	Very bad	1.1626	0.471	3.198	(1.271–8.051)	0.014
Lipid-lowering treatment	Yes	−0.4613	0.126	0.630	(0.492–0.807)	<0.001
Diabetes treatment	Yes	0.6974	0.134	2.009	(1.545–2.612)	<0.001
Age	(years)	0.1459	0.015	1.157	(1.124–1.192)	<0.001
Main daily act. × T		−0.0098	0.004	0.990	(0.982–0.998)	0.007
Age: Other causes		−0.0469	0.017	0.954	(0.923–0.986)	0.005

n sample train = 2201; deaths due to cardiovascular causes = 149; deaths due to other causes = 317; n sample test = 856; Likelihood Ratio Test (Chi2 = 821.8; *p* < 0.001). C-index testing = 0.773 (95%CI 0.740–0.805).

**Table 3:** Cardiovascular mortality risk scale for women aged 50 years and older with at least one cardiovascular risk factor and no previous cardiovascular event.

Variables		Score
Age	50–54	−2
	55–59	−1
	60–64	0
	65–69	1
	70–74	2
	75–79	3
	80–84	4
	85–89	5
	90–94	6
	95–99	7
	100–105	8
Main daily activity	Some activity	0
	Sitting most of the time	2
Physical activity leisure	Some activity	0
	Sedentary	1
Consumption of legumes	On a daily basis	0
	1–6 times/week	2
	Never or almost never	3
Self-perceived health	Very good	0
	Good, fair or bad	1
	Very bad	2
Dyslipidaemia treatment	No	0
	Yes	−1
Diabetes treatment	No	0
	Yes	1
Sum of Points	Risk	Mean Survival Time in Years (95% CI)
−1	0.0%	11.1 (9.9–12.3)
0	0.0%	9.5 (9.2–9.8)
1	0.0%	8.8 (8.7–8.9)
2	0.0%	8.0 (7.9–8.1)
3	0.1%	7.1 (7.0–7.2)
4	0.2%	6.4 (6.3–6.5)
5	0.4%	5.5 (5.4–5.6)
6	0.7%	4.7 (4.6–4.8)
7	1.5%	4.0 (3.9–4.1)
8	3.2%	3.2 (3.1–3.3)
9	6.5%	2.6 (2.5–2.7)
10	13.0%	2.0 (1.9–2.1)
11	25.1%	1.5 (1.4–1.6)
12	45.0%	1.2 (1.0–1.4)
13	71.1%	0.8 (0.6–1.0)
14	92.4%	0.6 (0.3–0.9)
15	>99%	0.0

## Data Availability

The data supporting the reported results in this study are publicly available on the website of the 2011 Spanish National Health Survey (ENSE11) and can be accessed at https://www.sanidad.gob.es/estadEstudios/estadisticas/encuestaNacional/encuesta2011.htm (accessed on 1 August 2024). Data from the national mortality register are public. No new data were created during this study.

## Supplementary Materials

The following supporting information can be downloaded at: https://www.mdpi.com/article/10.3390/jcdd11090287/s1, Table S1: Description of the sample using population proportion estimates; Table S2. The adjusted risks of cardiovascular death estimated by competing risk models for each explanatory variable with a crude adjustment for age.
