# Supplementary material for: Lifestyle Habits and Risk of Cardiovascular Mortality in Menopausal Women with Cardiovascular Risk Factors: A Retrospective Cohort Study
Source: J Cardiovasc Dev Dis. 2024 Sep 16;11(9):287. doi: 10.3390/jcdd11090287 (PMC11432577; doi:10.3390/jcdd11090287)
Supplement: Supplementary file 1 [file jcdd-11-00287-s001.zip › jcdd-3176594-supplementary.pdf]

Table S1. Description of the sample using population proportion estimates.

Variables		n	%
Cardiovascular Mortality	Alive	2488	81.4%
	Cardiovascular	180	5.9%
	Other causes	389	12.7%
Autonomous Community	Andalucía	486	15.9%
	Aragón	93	3.0%
	Asturias, Principado de	83	2.7%
	Balears, Illes	56	1.8%
	Canarias	122	4.0%
	Cantabria	39	1.3%
	Castilla y León	184	6.0%
	Castilla-La Mancha	145	4.7%
	Cataluña	504	16.5%
	Comunitat Valenciana	360	11.8%
	Extremadura	72	2.4%
	Galicia	236	7.7%
	Madrid, Comunidad de	358	11.7%
	Murcia, Región de	85	2.8%
	Navarra, Comunidad Foral de	39	1.3%
	País Vasco	165	5.4%
	Rioja, La	20	0.7%
	Ceuta	5	0.2%
	Melilla	4	0.1%
Social Class	Social Class I	223	7.3%
	Social Class II	188	6.1%
	Social Class III	509	16.7%
	Social Class IV	453	14.8%
	Social Class V	980	32.1%
	Social Class VI	526	17.2%
	NS/NA	178	5.8%
Body Mass Index	Normal	788	25.8%
	Overweight	1021	33.4%
	Obesity	709	23.2%
	NS/NA	539	17.6%
Country of Birth	Spain	2853	93.3%
	Foreign	204	6.7%
Marital Status	Single	209	6.8%
	Married	1834	60.0%
	Widowed	872	28.5%
	Separated	59	1.9%
	Divorced	82	2.7%
Educational Level	University Education	195	6.4%
	Vocational Training	182	6.0%
	High School	204	6.7%
	Secondary Education	928	30.3%
	Primary Education or Less	1548	50.6%
Monthly Net Income	NS/NA	846	27.7%
	> 2251 €	235	7.7%
	1551-2250 €	370	12.1%
	1051-1550 €	516	16.9%
	801-1050 €	383	12.5%
	< 800 €	708	23.1%
Tobacco Use	Never	2456	80.4%
	Former smoker	294	9.6%

	Smoker	306	10.0%
Total Tobacco Exposure	Never	2617	85.6%
	< 1h / day	166	5.4%
	> 1h / day	275	9.0%
Alcohol Risk	0.0	2276	74.5%
	1.0	781	25.5%
Hours of Sleep	> 9h / day	214	7.0%
	7-9 h / day	1835	60.0%
	< 7 h / day	1008	33.0%
Main Daily Activity	Sitting most of the time	1191	39.0%
	Standing most of the time	1628	53.3%
	Walking with some weight	209	6.8%
	Tasks with effort	29	0.9%
Leisure Physical Activity	Sedentary	1574	51.5%
	Occasional physical activity	1227	40.1%
	Frequent physical activity	140	4.6%
	Sports training	116	3.8%
Breakfast	At home	2924	95.7%
	Away from home	98	3.2%
	I usually don't have breakfast	34	1.1%
Fruit Consumption	Daily	2456	80.4%
	> 3 times/week	338	11.1%
	1-2 times/week	151	4.9%
	< 1 time/week	50	1.6%
	Never or almost never	61	2.0%
Vegetable Consumption	Daily	1800	58.9%
	> 3 times/week	943	30.9%
	1-2 times/week	244	8.0%
	< 1 time/week	52	1.7%
	Never or almost never	18	0.6%
Legume Consumption	Daily	57	1.9%
	> 3 times/week	731	23.9%
	1-2 times/week	1847	60.4%
	< 1 time/week	339	11.1%
	Never or almost never	83	2.7%
Dairy Consumption	Daily	2707	88.6%
	> 3 times/week	169	5.5%
	1-2 times/week	77	2.5%
	< 1 time/week	43	1.4%
	Never or almost never	61	2.0%
Sweets Consumption	Daily	810	26.5%
	> 3 times/week	336	11.0%
	1-2 times/week	469	15.3%
	< 1 time/week	570	18.7%
	Never or almost never	872	28.5%
Fast Food Consumption	Daily	13	0.4%
	> 3 times/week	23	0.8%
	1-2 times/week	146	4.8%
	< 1 time/week	442	14.5%
	Never or almost never	2433	79.6%
Dental Hygiene	3 or more times/ day	1047	34.2%
	Twice / day	1008	33.0%
	Once / day	673	22.0%
	Never or occasionally	329	10.8%
Self-Perceived Health	Very good	190	6.2%
	Good	1151	37.7%

	Fair	1173	38.4%
	Poor	432	14.1%
	Very poor	111	3.6%
Chronic Disease	No	795	26.0%
	Yes	2262	74.0%
Varicose Veins	No	2082	68.1%
	Yes	975	31.9%
Osteoarthritis	No	1306	42.7%
	Yes	1751	57.3%
Chronic Neck Pain	No	1968	64.4%
	Yes	1089	35.6%
Chronic Low Back Pain	No	1875	61.3%
	Yes	1182	38.7%
Allergies	No	2655	86.9%
	Yes	402	13.1%
Asthma	No	2865	93.7%
	Yes	192	6.3%
Chronic Obstructive Pulmonary Disease (COPD)	No	2835	92.7%
	Yes	222	7.3%
Stomach Ulcer	No	2873	94.0%
	Yes	184	6.0%
Urinary Incontinence	No	2774	90.7%
	Yes	283	9.3%
Cataracts	No	2325	76.1%
	Yes	732	23.9%
Skin Problems	No	2859	93.5%
	Yes	198	6.5%
Constipation	No	2735	89.5%
	Yes	322	10.5%
Cirrhosis	No	3014	98.6%
	Yes	43	1.4%
Depression	No	2458	80.4%
	Yes	599	19.6%
Anxiety	No	2539	83.1%
	Yes	518	16.9%
Other Mental Health Issues	No	2985	97.6%
	Yes	72	2.4%
Migraine	No	2534	82.9%
	Yes	523	17.1%
Hemorrhoids	No	2683	87.8%
	Yes	374	12.2%
Tumors	No	2859	93.5%
	Yes	198	6.5%
Osteoporosis	No	2530	82.8%
	Yes	527	17.2%
Thyroid Problems	No	2655	86.8%
	Yes	402	13.2%
Accident-Related Injuries	No	2887	94.4%
	Yes	170	5.6%
Accidents in the Last Year	No	2775	90.8%
	Yes	282	9.2%
Glasses/Contact Lenses Use	No	335	10.9%
	Yes	2722	89.1%
Hearing Aid Use	No	2865	93.7%
	Yes	192	6.3%
	No psychopathology	2420	79.2%

Mental Health (GHQ-12 Total Score)	Suspected	205	6.7%
	Psychopathology present	363	11.9%
	NS/NA	69	2.3%
Activity Limitation for Six Months	Severely limited	211	6.9%
	Midly limited	962	31.5%
	Not limited	1884	61.6%
Activity Limitation in the Last Two Weeks	No	2518	82.4%
	Yes	539	17.6%
Bedridden in the Last Two Weeks	No	2821	92.3%
	Yes	236	7.7%
Hospital Admission in the Last Yea	No	2781	91.0%
	Yes	276	9.0%
Primary Care Visit in the Last Month	No	1625	53.1%
	Yes	1432	46.9%
Specialist Visit in the Last Month	No	2418	79.1%
	Yes	639	20.9%
Day Hospital Visit in the Last Year	No	2771	90.6%
	Yes	286	9.4%
Emergency Visit in the Last Year	No	2212	72.4%
	Yes	845	27.6%
Physiotherapist Visit in the Last Year	No	2740	89.6%
	Yes	317	10.4%
Psychologist Visit in the Last Year	No	2910	95.2%
	Yes	147	4.8%
X-Ray in the Last Year	No	1916	62.7%
	Yes	1141	37.3%
CT Scan in the Last Year	No	2739	89.6%
	Yes	318	10.4%
Ultrasound in the Last Year	No	2566	83.9%
	Yes	491	16.1%
MRI in the Last Year	No	2767	90.5%
	Yes	290	9.5%
Flu Vaccine	No	1686	55.1%
	Yes	1371	44.9%
Treatment for Hypertension	No	1286	42.1%
	Yes	1771	57.9%
Treatment for Cholesterol	No	1912	62.6%
	Yes	1145	37.4%
Treatment for Diabetes	No	2557	83.7%
	Yes	500	16.3%
Age - Mean (SD)	(years)	67.9	(10.6)
EQ-5D Visual Analog Scale (VAS) - Mean (SD)	(0-100)	66.0	(19.5)

NS/NA: Not Specified/Not Answered

Table S2. The adjusted risks of cardiovascular death estimated by competing risk models for each explanatory variable with a crude adjustment for age.

Variables		HR	95% CI	p-value
SOCIODEMOGRAPHIC VARIABLES				
Social Class	Social Class I	1		
	Social Class II	0.596	(0.301-1.181)	0.139
	Social Class III	0.781	(0.473-1.290)	0.334
	Social Class IV	0.786	(0.466-1.327)	0.368
	Social Class V	0.931	(0.579-1.496)	0.767
	Social Class VI	0.824	(0.506-1.342)	0.437
	NS/NA	0.738	(0.429-1.270)	0.272
Body Mass Index (BMI)	Normal	1		
	Overweight	0.953	(0.717-1.266)	0.740
	Obesity	1.427	(1.051-1.937)	0.022
	NS/NA	1.234	(0.936-1.627)	0.135
Country of Birth	Spain	1		
	Foreign	0.996	(0.465-2.135)	0.992
Marital Status	Single			
	Married	0.692	(0.459-1.042)	0.077
	Widowed	0.709	(0.470-1.070)	0.101
	Separated	0.557	(0.223-1.392)	0.210
	Divorced	0.760	(0.280-2.061)	0.590
Educational Level	University Education	1		
	Vocational Training	0.537	(0.217-1.331)	0.179
	High School	1.381	(0.663-2.874)	0.388
	Secondary Education	1.405	(0.804-2.456)	0.232
	Primary Education or Less	1.562	(0.927-2.631)	0.094
Monthly Net Income	NS/NA	1		
	>1550 €	1.424	(0.835-2.427)	0.194
	1051-1550 €	1.080	(0.702-1.662)	0.727
	801-1050 €	1.104	(0.802-1.519)	0.545
	< 800 €	1.084	(0.779-1.510)	0.632
LIFESTYLE HABITS				
Tobacco Use	Never	1		
	Former smoker	1.417	(0.924-2.172)	0.110
	Smoker	2.087	(1.286-3.386)	0.003
Total Tobacco Exposure	Never	1		
	< 1h / day	1.275	(0.770-2.110)	0.345
	> 1h / day	1.342	(0.876-2.058)	0.176
Alcohol Risk	Does not drink	1		
	Drink	0.749	(0.580-0.966)	0.027
Hours of Sleep	7-9 h / day	1		
	< 7 h / day	1.246	(1.014-1.531)	0.035
	> 9h / day	1.676	(1.230-2.284)	0.001
Main Daily Activity	Sitting most of the time	1		
	Standing most of the time	0.478	(0.382-0.599)	<0.001
	Walking with some weight	0.460	(0.229-0.926)	0.030
	Tasks with effort	0.451	(0.126-1.610)	0.220
Leisure Physical Activity	Sedentary	1		
	Occasional physical activity	0.520	(0.422-0.641)	<0.001
	Frequent physical activity	0.292	(0.136-0.624)	0.002
	Sports training	0.610	(0.300-1.240)	0.172

DIET				
Breakfast	At home	1		
	Away from home	0.899	(0.386-2.092)	0.805
	I usually don't have breakfast	0.974	(0.220-4.311)	0.972
Fruit Consumption	Daily	1		
	> 3 times/week	1.064	(0.798-1.420)	0.672
	1-2 times/week	1.572	(0.975-2.536)	0.064
	< 1 time/week	0.991	(0.460-2.137)	0.981
	Never or almost never	1.235	(0.595-2.566)	0.572
Vegetable Consumption	Daily	1		
	> 3 times/week	1.086	(0.884-1.334)	0.433
	1-2 times/week	1.541	(1.109-2.142)	0.010
	< 1 time/week	1.413	(0.782-2.555)	0.252
	Never or almost never	1.393	(0.682-2.843)	0.363
Legume Consumption	Daily	1		
	> 3 times/week	1.872	(0.693-5.056)	0.216
	1-2 times/week	2.041	(0.763-5.459)	0.155
	< 1 time/week	2.282	(0.835-6.236)	0.108
	Never or almost never	3.027	(1.038-8.825)	0.042
Dairy Consumption	Daily	1		
	> 3 times/week	0.849	(0.538-1.341)	0.484
	1-2 times/week	0.851	(0.424-1.706)	0.649
	< 1 time/week	2.171	(0.867-5.432)	0.097
	Never or almost never	0.726	(0.373-1.413)	0.346
Sweets Consumption	Daily	1		
	> 3 times/week	1.188	(0.829-1.704)	0.347
	1-2 times/week	1.278	(0.951-1.718)	0.103
	< 1 time/week	1.097	(0.797-1.510)	0.569
	Never or almost never	1.468	(1.149-1.876)	0.002
Fast Food Consumption	Daily			
	> 3 times/week	-		
	1-2 times/week	-		
	< 1 time/week	-		
	Never or almost never	-		
Dental Hygiene	3 or more times/ day	1		
	Twice / day	1.238	(0.948-1.616)	0.117
	Once / day	1.279	(0.983-1.663)	0.067
	Never or occasionally	1.760	(1.317-2.352)	<0.001
CHRONIC HEALTH CONDITIONS				
Self-Perceived Health	Very good	1		
	Good	1.900	(1.005-3.593)	0.048
	Fair	2.731	(1.462-5.104)	0.002
	Poor	3.647	(1.906-6.977)	<0.001
	Very poor	5.763	(2.796-11.878)	<0.001
EQ-5D Visual Analog Scale (VAS)	(0-100)	0.982	(0.976-0.987)	<0.001
Chronic Disease	Yes	1.404	(1.109-1.776)	0.005
Varicose Veins	Yes	1.052	(0.864-1.279)	0.616
Osteoarthritis	Yes	1.055	(0.864-1.288)	0.600
Chronic Neck Pain	Yes	1.152	(0.943-1.407)	0.165

Chronic Low Back Pain	Yes	1.237	(1.018-1.501)	0.033
Allergies	Yes	1.116	(0.848-1.469)	0.432
Asthma	Yes	1.863	(1.307-2.657)	0.001
Chronic Obstructive Pulmonary Disease (COPD)	Yes	1.712	(1.208-2.427)	0.003
Stomach Ulcer	Yes	1.001	(0.677-1.482)	0.995
Urinary Incontinence	Yes	1.446	(1.134-1.844)	0.003
Cataracts	Yes	0.908	(0.742-1.111)	0.352
Skin Problems	Yes	1.516	(1.000-2.296)	0.050
Constipation	Yes	1.324	(1.010-1.735)	0.042
Cirrhosis	Yes	1.939	(0.986-3.812)	0.055
Depression	Yes	1.216	(0.959-1.542)	0.106
Anxiety	Yes	1.149	(0.873-1.512)	0.320
Other Mental Health Issues	Yes	2.810	(1.902-4.150)	<0.001
Migraine	Yes	1.011	(0.779-1.312)	0.935
Hemorrhoids	Yes	0.909	(0.663-1.246)	0.555
Tumors	Yes	1.426	(1.012-2.009)	0.043
Osteoporosis	Yes	1.035	(0.813-1.317)	0.777
Thyroid Problems	Yes	0.855	(0.601-1.217)	0.384
Accident-Related Injuries	Yes	0.940	(0.584-1.513)	0.799
Accidents in the Last Year	Yes	1.394	(1.053-1.845)	0.020
Glasses/Contact Lenses Use	Yes	0.690	(0.527-0.905)	0.007
Hearing Aid Use	Yes	1.009	(0.746-1.365)	0.952
MENTAL HEALTH AND DISABILITY				
Mental Health (GHQ-12 Total Score)	No psychopathology	1		
	Suspected	1.202	(0.821-1.757)	0.343
	Psychopathology present	1.988	(1.535-2.574)	<0.001
	NS/NA	2.660	(1.762-4.014)	<0.001
Activity Limitation for Six Months	Severely limited	1		
	Midly limited	0.482	(0.361-0.643)	<0.001
	Not limited	0.326	(0.244-0.437)	<0.001
Activity Limitation in the Last Two Weeks	Yes	1.699	(1.351-2.137)	<0.001
Bedridden in the Last Two Weeks	Yes	1.997	(1.406-2.836)	<0.001
HEALTHCARE SERVICES UTILIZATION				
Hospital Admission in the Last Year	Yes	1.962	(1.494-2.576)	<0.001
Primary Care Visit in the Last Month	Yes	1.092	(0.905-1.318)	0.361
Specialist Visit in the Last Month	Yes	1.575	(1.255-1.977)	<0.001

Day Hospital Visit in the Last Year	Yes	1.167	(0.843-1.616)	0.351
Emergency Visit in the Last Year	Yes	1.416	(1.157-1.732)	0.001
Physiotherapist Visit in the Last Year	Yes	0.984	(0.676-1.434)	0.935
Psychologist Visit in the Last Year	Yes	1.172	(0.759-1.811)	0.475
X-Ray in the Last Year	Yes	1.231	(1.010-1.500)	0.041
CT Scan in the Last Year	Yes	2.000	(1.499-2.668)	<0.001
Ultrasound in the Last Year	Yes	1.311	(0.991-1.736)	0.057
MRI in the Last Year	Yes	1.256	(0.874-1.805)	0.216
Flu Vaccine	Yes	1.048	(0.855-1.285)	0.652
TREATMENTS				
Treatment for Hypertension	Yes	1.066	(0.870-1.307)	0.540
Treatment for Cholesterol	Yes	0.724	(0.593-0.885)	0.002
Treatment for Diabetes	Yes	1.899	(1.549-2.329)	<0.001

NS/NA: Not Specified/Not Answered
